# Diagnostic Evaluation of the IS1081-Targeted Real-Time PCR for Detection of *Mycobacterium bovis* DNA in Bovine Milk Samples

**DOI:** 10.3390/pathogens12080972

**Published:** 2023-07-25

**Authors:** Mohamed M. Zeineldin, Kimberly Lehman, Patrick Camp, David Farrell, Tyler C. Thacker

**Affiliations:** National Veterinary Services Laboratories, Veterinary Services, Animal and Plant Health Inspection Service, United States Department of Agriculture, Ames, IA 50010, USA; mohamed.zeineldin@usda.gov (M.M.Z.);

**Keywords:** *Mycobacterium bovis*, PCR, IS1081, milk, PrimeStore^®^ MTM, diagnostics

## Abstract

The ability of *Mycobacterium bovis* (*M. bovis*) to survive in bovine milk has emerged as a serious public health concern. The first objective of this study was to evaluate the diagnostic utility of IS1081-targeted real-time PCR for the detection of *M. bovis* DNA in different fractions of bovine milk. In a model study, bovine milk samples were spiked with serially diluted *M. bovis* BCG to investigate the detection limit of *M. bovis* DNA in whole milk and milk fractions (cream, pellet, and pellet + cream combined) using IS1081 real-time PCR. The assay was then used to detect *M. bovis* DNA in whole milk and milk fractions from naturally infected animals. The results showed that the IS1081 real-time PCR was more sensitive when detecting *M. bovis* DNA in the cream layer alone and cream + pellet combined compared to whole milk or the pellet alone. While PCR-based diagnostic assays for the detection of *M. bovis* in milk samples provide a quicker diagnostic tool for bovine tuberculosis, safe processing, and handling of *M. bovis*-infected milk samples remain a challenge and pose a human health risk. PrimeStore Molecular Transport Medium (MTM) has been shown to rapidly inactivate infected specimens while preserving nucleic acid for subsequent Molecular analysis. Therefore, the secondary objective of this study was to evaluate the ability of MTM to inactivate *M. bovis* BCG in spiked milk samples as well as its ability to preserve BCG DNA for the PCR assay. The results showed that MTM can successfully inactivate BCG alone or in spiked milk samples while preserving DNA for the PCR assay. The CT values of *M. bovis* BCG alone and spiked milk samples aliquoted in MTM and without MTM were similar at various dilutions. Taken together, our results indicate that using DNA extracted from the milk cream fraction alone or combined milk cream and pellet improved the recovery rate of *M. bovis* DNA in bovine milk samples. MTM has the potential to provide a safe and rapid sample processing tool for *M. bovis* inactivation in milk samples and preserve DNA for molecular diagnostics.

## 1. Introduction

Bovine tuberculosis (bTB), caused predominantly by *Mycobacterium bovis* (*M. bovis*), is a chronic granulomatous infectious disease affecting a wide range of animal species and can lead to significant economic loss [1,2]. The ability of *M. bovis* to survive in bovine milk and dairy products has emerged as a serious public health concern, particularly among individuals who consume raw milk and unpasteurized dairy products [3,4]. *M. bovis* bacilli have been found to survive in cream cheese and yogurt produced from raw milk for up to 14 days and can persist in butter for up to 100 days [5,6]. While widespread milk pasteurization and strict hygiene measures in livestock management have greatly reduced the transmission of bTB in developed countries, it is challenging to quantify the risk of tuberculosis associated with raw milk due to insufficient studies on consumption patterns [7]. Currently, several methods are available for the detection of *M. bovis* in milk and dairy products, including bacterial isolation [8,9,10]. Additionally, microscopic detection of *M. bovis* in milk can be accomplished quickly by direct staining of milk samples; but this methodology lacks sensitivity and specificity [11]. Although *M. bovis* culture is considered the gold-standard diagnostic method, it requires a high bacterial load and is time-consuming, taking up to eight weeks for a definitive diagnosis [12]. To overcome these challenges, various molecular techniques, such as polymerase chain reaction (PCR) and its variants (simplex PCR, multiplex PCR, nested PCR, real-time PCR, and nested real-time PCR), have been developed for quick and specific identification of *M. bovis* in milk samples [13,14,15,16,17,18]. The diagnostic performance of PCR-based assays has been evaluated as a potential first-line technique for detecting *M. bovis* in milk samples, using microbiological culture as a reference [14].

In comparison to microbiological culture, PCR-based methods can be affected by variations in the levels of target DNA in milk samples, PCR reagents, and DNA extraction methods [19]. Additionally, milk is a complex biological fluid consisting mainly of fat globules, casein micelles, a relatively high number of somatic cells, bacterial flora, and calcium constituents that act as potential PCR inhibitors [20]. DNA extraction from milk samples for PCR testing is commonly performed by pelleting the casein and discarding the milk cream fraction, even though milk cream has been found to harbor relatively high numbers of microorganisms [21]. According to Poms et al. (2001), removing the milk cream fraction during the extraction procedure can result in a great loss of bacterial DNA, thus reducing PCR sensitivity [22]. Based on this information, the first objective of this study was to evaluate the diagnostic utility of a direct real-time PCR method targeting IS1081 to detect *M. bovis* DNA extracted from whole milk and milk fractions (cream, pellet, and pellet + cream combined).

Currently, various techniques have been evaluated for inactivating *M. bovis* from diagnostic specimens and cultures, including heat, chemicals, or a combination of methods [23,24]. However, such procedures before DNA extraction can lead to a loss of bacterial DNA and impact PCR sensitivity. Therefore, exploring alternative approaches to rapidly inactivate infected samples while preserving DNA for downstream molecular diagnostics is necessary. PrimeStore^®^ MTM (PrimeStore, Longhorn Vaccines & Diagnostics, Austin, TX, USA) is a commercial medium used for sample collection, storage, and transport at ambient temperature [25]. Prime Store MTM is composed of an optimized blend of chelating, chaotrophic detergents, and electrolyte reagents specifically formulated for sample inactivation, lysis of biological pathogens, and stabilizing and preserving released nucleic acids for a prolonged time. Previous studies have shown that MTM effectively inactivated several viruses, fungi, and bacteria, including *M. tuberculosis*, and preserved nucleic acids at ambient temperature for downstream molecular testing [26,27,28]. Based on the potential utility of MTM for collection, transportation, and inactivation of potentially infectious pathogens, the secondary objective of this study was to evaluate the ability of MTM to inactivate *M. bovis* BCG, as well as to preserve BCG DNA for PCR-based diagnostics.

## 2. Materials and Methods

### 2.1. Preparation of M. bovis BCG Stock

In this study, *M. bovis* BCG, a vaccine strain, was grown on Middlebrook 7H11 agar slants and then suspended in Middlebrook 7H9 broth. BCG cells were harvested from a Middlebrook 7H9 broth at mid- to late-logarithmic growth (3-week-old broth). The harvested cells were aliquoted into vials (1.5 mL aliquots) and frozen at −80 °C. Dilutions of BCG were prepared by making 10-fold serial dilutions of the frozen BCG stock in PBS (ranged from undiluted *M. bovis* BCG to 1:10^10^ dilution). A total of 100 μL of each dilution was transferred to a DNA extraction bead tube. DNA was extracted from the serially diluted BCG using the MagMAX™ Total Nucleic Acid Isolation Kit (96 well plate format, Thermo Fisher Scientific, Waltham, MA, USA) according to the manufacturer’s instructions. DNA was quantified using a Qubit^®^ fluorometer (Thermo Fisher Scientific, Waltham, MA, USA) according to manufacturer’s instructions. The CT value of *M. bovis* BCG DNA from the serially diluted frozen aliquots was determined using IS1081 targeted real-time PCR.

### 2.2. Preparation of Spiked Milk Samples, DNA Extraction, and Real-Time PCR

Milk samples from a bTB-free herd were used to determine the assay limit of detection (LOD). One mL of diluted BCG (ranged from undiluted *M. bovis* BCG to 1:10^10^ dilution) was added to 9 mL of whole milk to generate spiked milk samples containing BCG. A total of 100 μL of spiked whole milk was transferred to a DNA extraction bead tube (2-mL screwcap tube containing 100 µL 0.1 mm glass beads, 400 µL TE buffer, and Biolin internal control). To assess milk fractions, 1.5 mL of the spiked milk samples were aliquoted into 2 mL Eppendorf tubes and fractionated by centrifugation at 5500× *g* for 10 min at 4 °C. For DNA extraction from the milk cream, the whole cream layer was transferred using a sterile loop to a DNA extraction bead tube. For DNA extraction from the milk pellet, the cream layer and whey were carefully discarded, and the remaining pellet was suspended in 100 μL PBS and then transferred to the DNA extraction bead tube. For DNA extraction from milk cream + pellet combined, the whey fraction was carefully removed, and cream and pellet contents were combined and transferred to a DNA extraction bead tube. For the positive control, 100 μL of each BCG stock dilution was added to a DNA extraction bead tube. Each individual fraction and control were then heat-killed for 30 min at 105 °C. DNA was extracted from the spiked whole milk and milk fractions (cream only, pellet only, and cream + pellet combined) using the MagMAX™ Total Nucleic Acid Isolation Kit (96 well plate format, Thermo Fisher Scientific, MA, USA) according to the manufacturer’s instructions. Reference whole milk and milk sample fractions (i.e., unspiked milk) were included for DNA extraction as controls. The CT value of the *M. bovis* DNA from whole milk and milk fractions was determined using IS1081 real-time PCR assay with the ViiA7 real-time PCR system (Thermo Fisher Scientific, Waltham, MA, USA). The PCR run was considered valid if the positive control and internal control performed as expected. The analytical specificity of the IS1081 primer was previously assessed in our laboratory [29].

### 2.3. Clinical Milk Samples from Naturally Infected Animals

The diagnostic performance of the IS1081 targeted real-time PCR was then assessed using three *M. bovis*-positive milk samples from naturally infected animals from dairy herds which had a history of *M. bovis* infection. Samples were randomly selected from milk samples that were submitted for routine testing at the National Veterinary Services Laboratories after routine diagnostic tests had been completed. Four replicates of selected milk samples were fractionated by centrifugation at 5500× *g* for 10 min at 4 °C as described above. The whole milk sample and milk fractions were heat inactivated for 30 min at 105 °C. DNA was extracted and PCR was performed as described above.

### 2.4. Inactivation of M. bovis in PrimeStore^®^ MTM

The ability of MTM to inactivate *M. bovis* was assessed for both BCG alone and BCG-spiked milk samples. Dilutions of BCG were prepared by making 10-fold serial dilutions of the frozen BCG in PBS (ranged from undiluted *M. bovis* BCG to 1:10^3^ dilution, 100 μL BCG was added to 900 μL of PBS). A total of 500 μL of serially diluted BCG and 500 μL of spiked milk samples at different dilutions were pipetted into 1.5 mL of MTM at a ratio of 1:3, sample-to-MTM. Samples were then mixed by vortexing and refrigerated overnight. Sample aliquots of 100 μL from BCG and spiked milk samples inoculated in MTM tubes were plated on Middlebrook 7H11 agar. A positive control was included (BCG and spiked milk samples without MTM plated on Middlebrook 7H11 agar). Effective inactivation of *M. bovis* at different dilutions was defined as no growth in all samples after 8 weeks of incubation.

### 2.5. Molecular Evaluation of Samples Preserved in MTM Tubes

Total genomic DNA from BCG and spiked milk samples in MTM and without MTM was extracted as described above. PCR detection of *M. bovis* in all samples was determined using the IS1081 real-time PCR assay as described above. The PCR run was considered valid if all controls performed as expected. Each sample was tested in triplicate to obtain an average CT.

### 2.6. Statistical Analysis

To evaluate the diagnostic utility of the IS1081 targeted real-time PCR method to detect *M. bovis* DNA in whole milk and milk fractions, the CT values of *M. bovis* DNA among different milk fractions were compared by a one-way analysis of variance (ANOVA) test. To evaluate the ability of MTM to preserve *M. bovis* DNA for molecular testing, the student’s *t*-test was used to compare the CT values of *M. bovis* DNA from samples aliquoted in MTM and without MTM. All statistical analyses were performed using JMP Pro 13 (SAS Institute Inc., Hong Kong, China), and the level of significance was determined at *p*-value ≤ 0.05.

## 3. Results

### 3.1. Detection of M. bovis DNA in Milk Sample Fractions

Four replicates of a 10-fold dilution series of *M. bovis* BCG (ranging from undiluted *M. bovis* BCG to 1:10^10^ dilution) were tested to determine the limit of detection of BCG in spiked milk samples using the IS1081 targeted real-time PCR. Our results showed that the range of CT values of serially diluted BCG spanned from an average of 14.5 cycles (SE: 0.72) in undiluted samples to 36.25 cycles (SE: 0.35) in 1:10^10^ dilution (Figure S1). The lowest concentration at which all four replicates were detected was the 10^−5^ dilution (0.01 ng DNA) with an average CT value of 34.3 (Figure S1).

**Figure 1:**
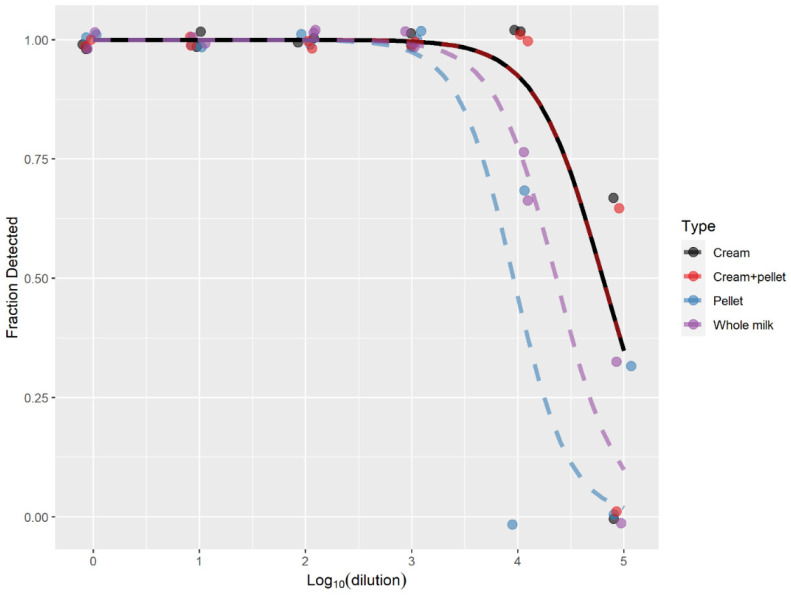
Fraction of spiked milk samples with serially diluted BCG detected using the IS1081 targeted direct real-time PCR. Seven replicates were tested for each dilution. The dashed lines are best-fit logistic curves.

**Figure 2:**
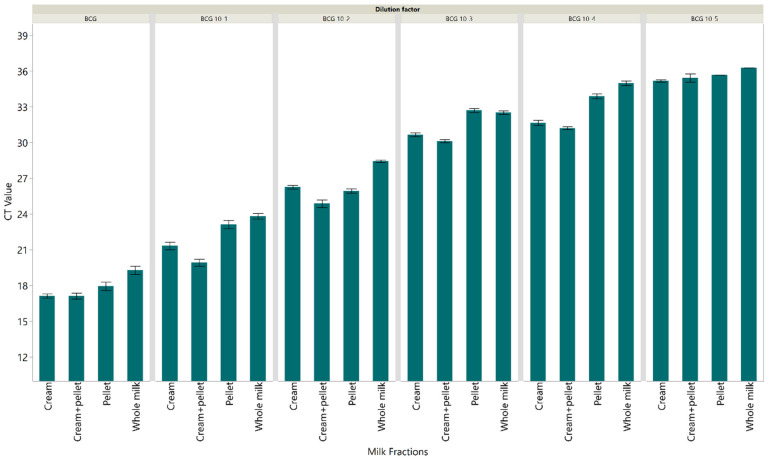
Cycle threshold values were detected using the IS1081 targeted direct real-time PCR for different fractions of milk samples spiked with serially diluted *M. bovis* BCG. Seven replicates were tested for each dilution. Each error bar represents the standard error from the mean.

To evaluate the diagnostic utility of the IS1081-targeted real-time PCR in the detection of *M. bovis* in whole milk and milk fractions, seven replicates of each dilution of the spiked milk samples were tested. The dilution at which all seven replicates were detected was 1:10^4^ ([0.1 ng *M. bovis* BCG DNA]) (Figure 1) for the cream and cream + pellet fractions. The extracted DNA from the cream fraction at 1:10^4^ yielded an average CT value of 31.7 (SE: 0.31) (Figure 2), while extracted DNA from the combined cream and pellet yielded an average CT value of 31.2 (SE: 0.41) (Figure 2). The dilution at which all seven replicates were detected for whole milk and pellet was the 1:10^3^ dilution (1 ng *M. bovis* BCG DNA) (Figure 1). The extracted DNA from the pellet fraction at 1:10^3^ yielded an average CT value of 32.7 (SE: 0.64) (Figure 2), while extracted DNA from the whole milk yielded an average CT value of 32.5 (SE: 0.18) (Figure 2). The results from the spiking experiment show that *M. bovis* in the cream layer alone and cream + pellet combined was easier to detect using the IS1081-targeted real-time PCR than *M. bovis* in whole milk or the pellet alone. Additionally, the real-time PCR analysis revealed no evidence of PCR inhibition, indicating that the amplification reactions proceeded efficiently and without interference in all tested samples and conditions.

### 3.2. Milk Samples from Naturally Infected Animals 

**Figure 3:**
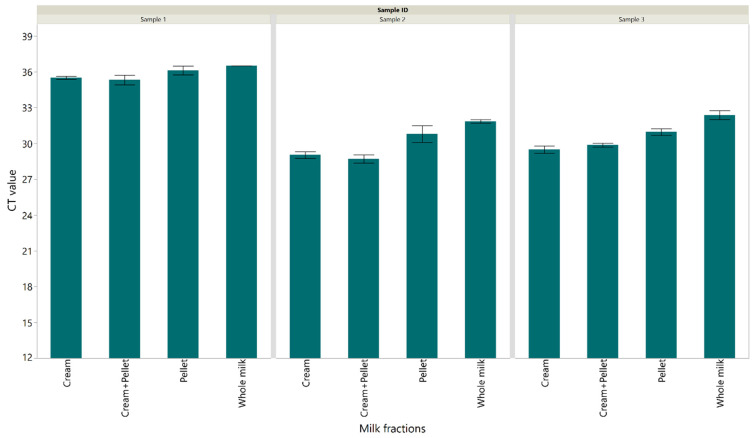
Cycle threshold values detected using the IS1081 targeted direct real-time PCR for different fractions of milk samples from naturally infected cows. Each error bar represents the standard error from the mean.

The diagnostic performance of the IS1081-targeted real-time PCR was assessed in milk from naturally infected animals from dairy herds with a history of *M. bovis* infection. Our results showed that *M. bovis* in the cream layer alone (an average CT value of 31.34) and cream + pellet combined (an average CT value of 31.29) were detected at a lower CT than *M. bovis* in whole milk (an average CT value of 32.6) or the pellet alone (an average CT value of 32.3) (Figure 3).

### 3.3. Inactivation of M. bovis BCG in PrimeStore^®^ MTM

**Figure 4:**
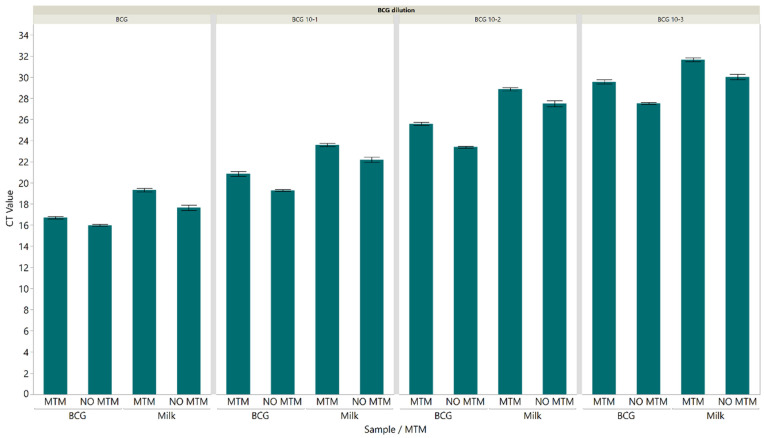
Cycle threshold values were detected using the IS1081 targeted direct real-time PCR for samples aliquoted in MTM and without MTM. There was no significant change in CT value detected among samples aliquoted in MTM and without MTM at different dilutions. Each error bar represents the standard error from the mean.

To assess the ability of MTM to inactivate BCG, serially diluted BCG and spiked milk samples at different dilutions were added in triplicate to MTM at a ratio of 1:3 (sample-to-MTM). Samples were stored at 4 °C overnight, then cultured on Middlebrook 7H11 agar and incubated at 37 °C for 8 weeks. There was no mycobacterial growth of BCG at any dilution after the 8 weeks incubation. In contrast, mycobacterial colonies were observed after 2–4 weeks incubation in the untreated dilutions. DNA from *M. bovis* BCG and spiked milk samples in MTM and without MTM was extracted and IS1081-targeted real-time PCR was then carried out to assess the ability of MTM to preserve the *M. bovis* BCG DNA. Our results showed that there was no significant change in CT value detected among samples aliquoted in MTM and without MTM at different dilutions (Figure 4).

## 4. Discussion

Bovine tuberculosis, caused by *M. bovis*, is a chronic zoonotic disease affecting a wide range of mammalian species, including humans [30]. Milk and dairy products from *M. bovis*-infected animals are a global zoonotic health concern and have significant economic implications for both the human and livestock industries [31]. While eradication programs along with widespread milk pasteurization have considerably reduced the prevalence of bTB in developed countries, the consumption of unpasteurized products still represents a major risk factor among individuals in developing countries [10,32]. Therefore, accurate, reliable, and cost-effective diagnostics are essential for the effective detection of *M. bovis* and the subsequent eradication of bTB. Despite several diagnostic tools being available to assess the sensitivity (Se) and specificity (Sp) of *M. bovis* diagnostic tests, *M. bovis* detection in milk samples poses a challenge due to the intermittent release of *M. bovis* in milk [33]. Another factor that can hinder the detection of *M. bovis* in milk is the complex composition of the starting material that impairs the DNA extraction from milk samples (e.g., milk proteins, fat globules, divalent cations, and host epithelial cells) [4]. Despite these limitations, several studies have shown that PCR-based assays can detect *M. bovis* in milk samples with much lower concentrations of *M. bovis* compared to traditional culture methods [34,35,36]. Previously, PCR testing of milk samples was commonly performed using the milk pellet fraction and discarding the milk cream, although partitioning of bacteria into the milk cream layer following centrifugation has been known [21]. The presence of high numbers of bacteria in the milk cream fraction is attributed to antibody-mediated binding of bacteria to milk fat globules [37,38], although other binding mechanisms (e.g., lymphocyte mediated, hydrophobic, and lectin-sugar) have been suggested to play a major role [39,40]. Therefore, the first objective of this study was to evaluate the diagnostic utility of the IS1081-targeted real-time PCR for direct detection of *M. bovis* in whole milk and milk fractions.

The primer for IS1081 has been widely used in diagnostic tests for bTB and is proven to have a higher analytical sensitivity than the detection of IS6110, due to the multi-copy nature of the IS1081 target [41,42,43]. The specificity of the IS1081 primer has been previously confirmed by testing a large number of *Mycobacterium* species [29]. In the current study, the lowest detection limit of the IS1081 targeted direct real-time PCR using *M. bovis* BCG was 10^−5^ dilution (0.01 ng *M. bovis* BCG DNA) with an average CT value of 34.3 with all four *M. bovis* BCG replicates showing an amplification curve. In this experimental model, the detection limit of *M. bovis* in whole milk and milk fractions (cream, pellet, and cream + pellet combined) was evaluated. The IS1081 targeted direct real-time PCR was additionally evaluated for the detection of *M. bovis* in milk from naturally infected animals. Our results indicate that an appropriate detection limit of *M. bovis* in infected milk samples was achieved, and this method was able to detect the *M. bovis* DNA directly in naturally infected milk samples. Our results indicated that detecting *M. bovis* in the cream layer, either alone or in combination with the pellet, was easier compared to detecting it in whole milk or the pellet alone. However, it is important to acknowledge that milk fat can hinder the process of DNA extraction and serve as a carrier for PCR inhibitors, thereby affecting DNA amplification [21]. Additionally, various crucial factors such as centrifugation speed, bead beating process, different fat content, storage temperature, and storage time can also impact DNA recovery from milk cream [38]. Based on our study’s results, the fat content has minimal impact on DNA recovery and PCR amplification efficiency. These findings support the hypothesis that incorporating the milk cream fraction during DNA extraction enhances the detection of *M. bovis* DNA in milk samples. Therefore, it is crucial to prioritize the development and optimization of DNA extraction and amplification protocols specifically for milk cream, especially if there are concerns about the potential reduction in bacterial yield by discarding the cream fraction.

The secondary objective of this study was to evaluate the ability of MTM to inactivate BCG in spiked milk samples as well as its ability to preserve BCG DNA for PCR-based diagnostics. According to previous reports, PrimeStore^®^ MTM was an efficient storage and transport medium for the inactivation of bacteria, fungi, and viruses as well as stabilizing and preserving the nucleic acids for molecular testing [26,27,28]. In line with other studies, samples preserved in MTM showed no mycobacterial growth after 8-week incubation on solid media compared to the positive control where mycobacterial colonies were observed after 2–3 weeks incubation [44,45]. Additionally, the CT value of *M. bovis* BCG DNA from samples aliquoted in MTM and without MTM was similar at different dilutions (Figure 4).

## 5. Conclusions

In conclusion, our results indicate that using DNA extracted from the milk cream fraction alone or combined milk cream and pellet improves the recovery rate of *M. bovis* DNA in bovine milk samples. While microbiological culture remains necessary for *M. bovis* isolation and molecular characterization for epidemiological purposes, utilization of IS1081-targeted real-time PCR demonstrates great potential for identifying *M. bovis* DNA in bovine milk samples. This method’s quick turnaround time makes it a valuable substitute for microbiological culture, and its implementation strategy should be determined based on eradication programs. Additionally, PrimeStore^®^ MTM provides a safe and efficient method for sample processing, transport, and inactivation of *M. bovis* BCG as well as preserving DNA for molecular diagnostics.

## Data Availability

Not applicable.

## Supplementary Materials

The following supporting information can be downloaded at: https://www.mdpi.com/article/10.3390/pathogens12080972/s1, Figure S1: Cycle threshold values detected using the IS1081 targeted direct real-time PCR for serially diluted *M. bovis* BCG at different concentrations. Four replicates were tested for each dilution.
