# Supplementary figures and images for: Diagnostic Evaluation of the IS1081-Targeted Real-Time PCR for Detection of Mycobacterium bovis DNA in Bovine Milk Samples
Source: Pathogens. 2023 Jul 25;12(8):972. doi: 10.3390/pathogens12080972 (PMC10458061; doi:10.3390/pathogens12080972)

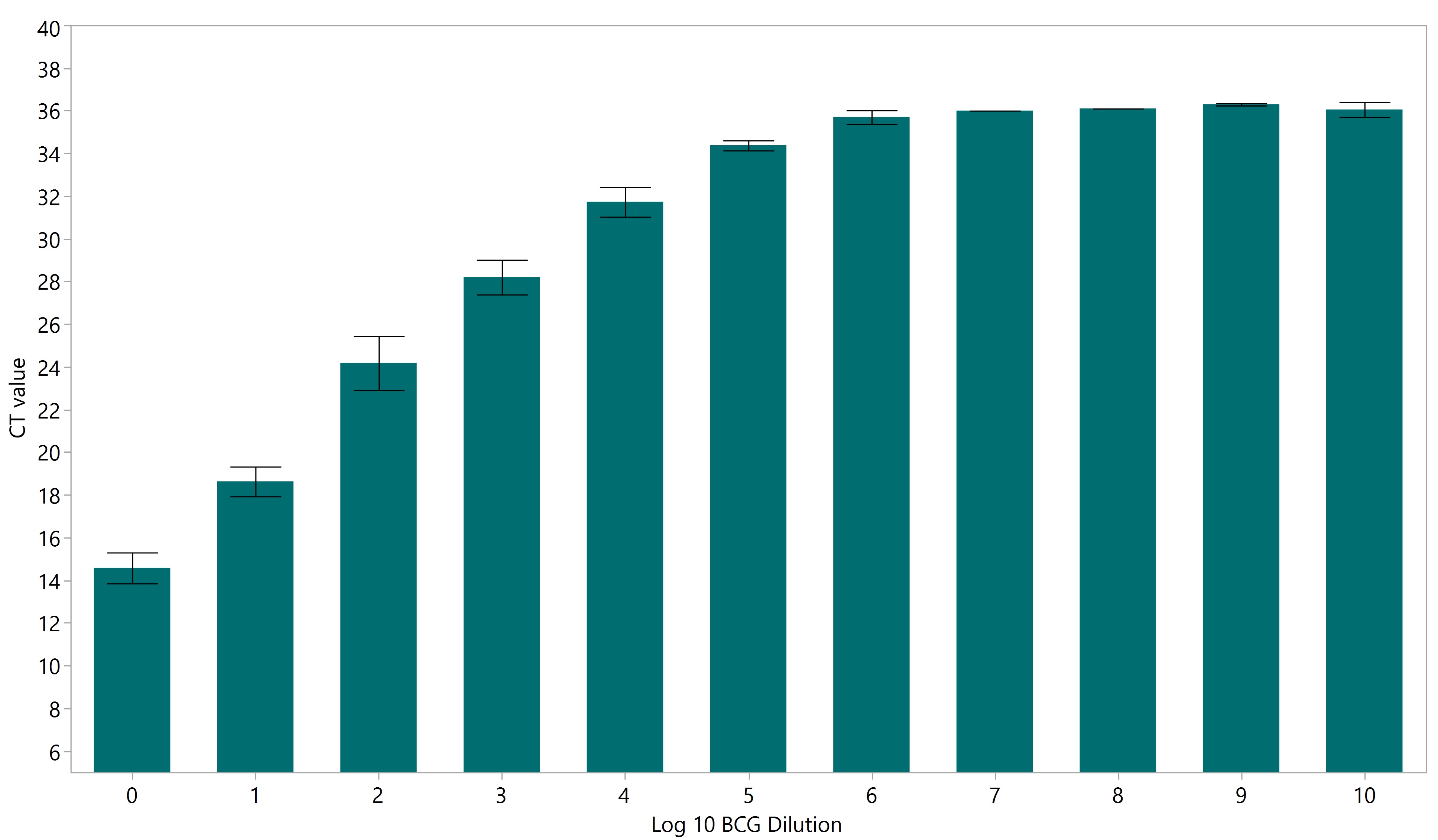

Supplement: Supplementary file 1 [file pathogens-12-00972-s001.zip › pathogens-2491268-Figure S1.jpg]
